# Supplementary material for: Wildfire Smoke Adjustment Factors for Low-Cost and Professional PM2.5 Monitors with Optical Sensors
Source: Sensors (Basel). 2020 Jun 30;20(13):3683. doi: 10.3390/s20133683 (PMC7374346; doi:10.3390/s20133683)
Supplement: Supplementary file 1 [file sensors-20-03683-s001.pdf]

30-June-2020

Supplementary Materials

Wildfire Smoke Adjustment Factors for Low-Cost and Professional $PM_{2.5}$ Monitors with Optical Sensors

William W. Delp and Brett C. Singer *

Indoor Environment Group and Residential Building Systems Group, Lawrence Berkeley National Laboratory, Berkeley, CA, USA

*Corresponding email: bcsinger@lbl.gov

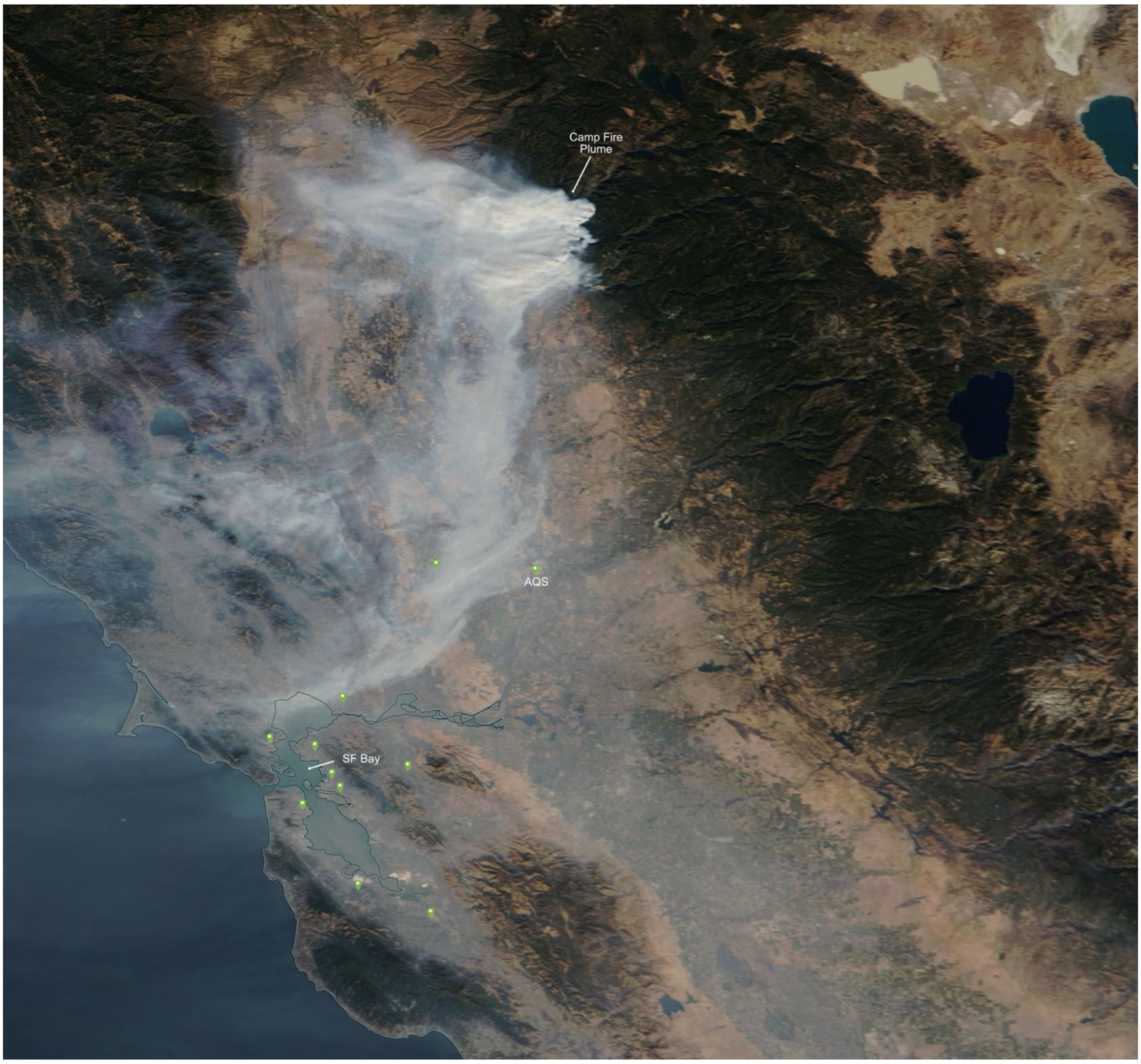

Figure S1. Satellite image of the Camp fire on 11/12/2018. (Extracted from the NASA Worldview application (https://worldview.earthdata.nasa.gov).

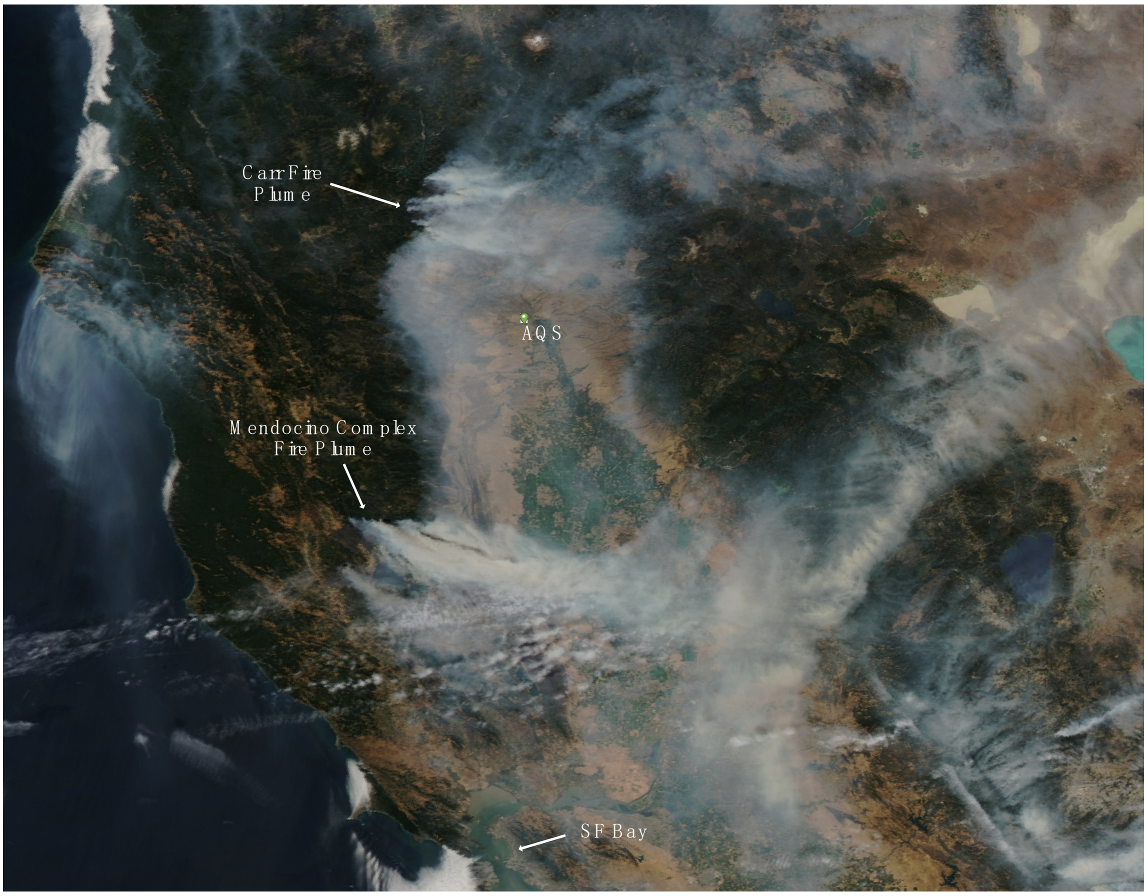

Figure S1. Satellite image of the Carr and Mendocino complex fires on 08/03/2018 20:43:47 GMT. (Extracted from the NASA Worldview application (https://worldview.earthdata.nasa.gov).

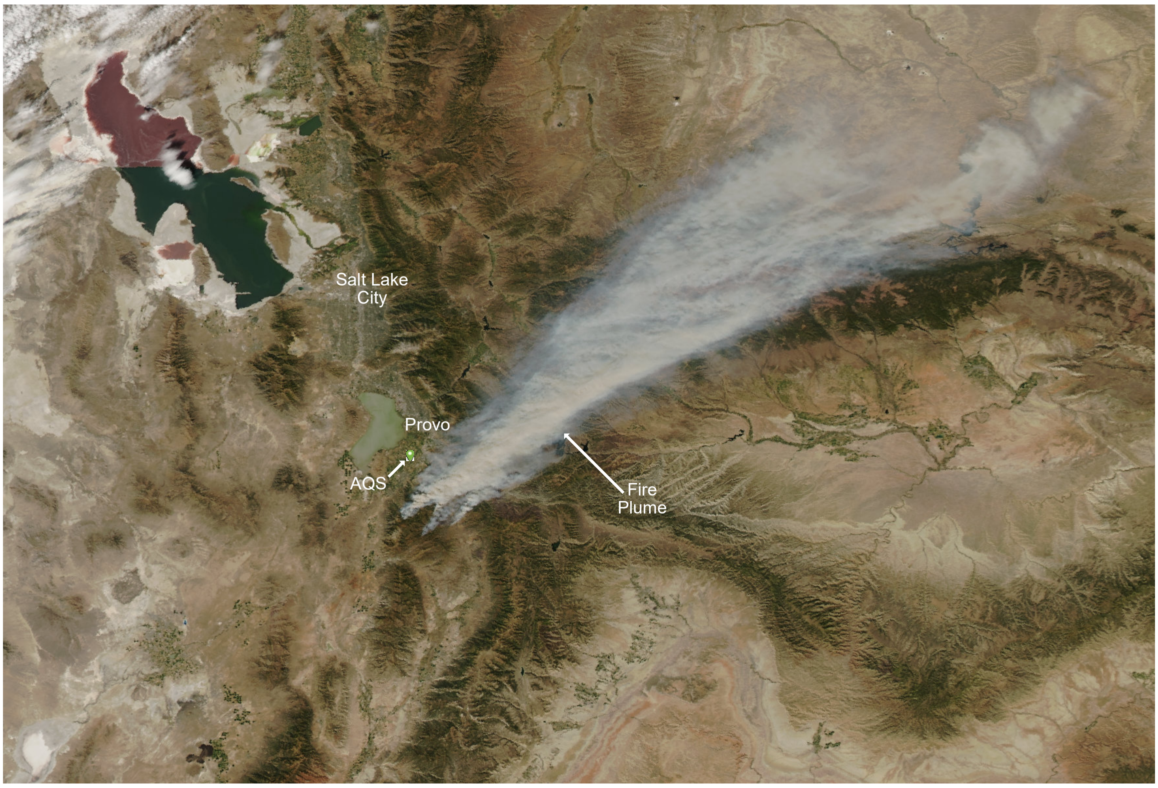

Figure S2. Satellite image of the Pole Creek fire 9/13/2018 20:36:57 GMT. (Extracted from the NASA Worldview application (https://worldview.earthdata.nasa.gov).

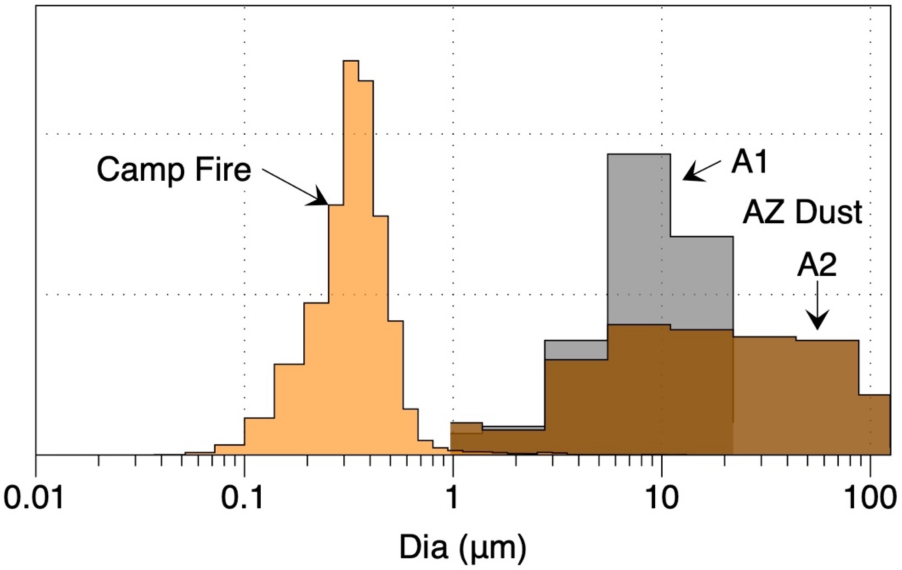

Figure S3. Mass distributions of wildfie smoke and two varieties of test dust.

Estimates of AERs during monitoring

In July–Aug 2017 we performed testing in the same laboratory space that was used for the co-location measurements of smoke from the Camp Fire. We measured ventilation rates in the space from 0.5 to over 1 air changes per hour (h^{-1}). The temperature differences inside to outside, defined as the measured room temperature minus the temperature measured at an on-site meteorological tower approximately 300m away, varied from 2.5 to over 10°C (median 7.2, IQR 5.3–8.3), in the summer of 2017, and from near 0 to 8°C (median 3.5, IQR 2.5–4.7) during the period of the Camp Fire. The actual driving forces were higher as there was a considerable solar load on two sides of the building along with the flat roof. Lower ventilation rates occurred at lower temperature differences. The peak solar loads (as measured at the meteorological tower) in November 2018 were typically between 430 and 510 w/m^2, while in summer 2017 they were between 480 and 900 w/m^2 on the days we measured the ventilation rate. The day with the lowest summer peak load (480 w/m^2) also had the lowest inside-outside temperature difference and the lowest measured air exchange rate of 0.5 h^{-1}. We thus estimate that the AER in the lab during the Camp Fire was at the low end of AERs measured in the summer of 2017.

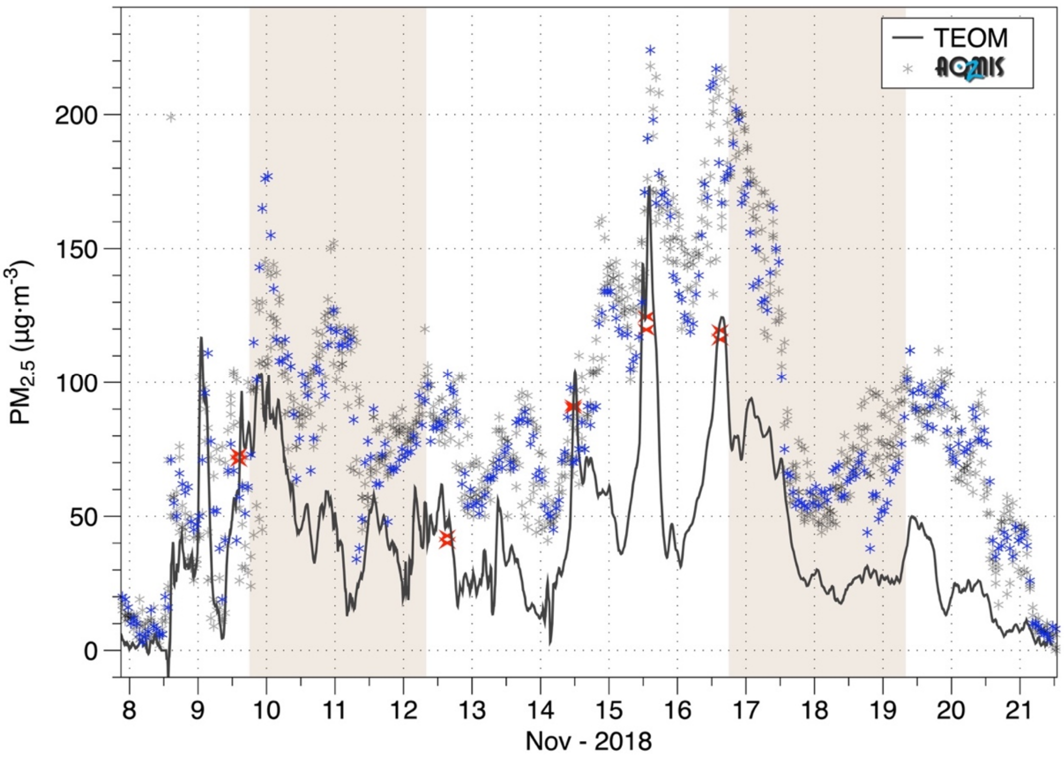

Figure S4. Time series of $PM_{2.5}$ measured by TEOM in the laboratory at LBNL lab and at the Berkeley and Oakland-West regulatory air quality monitoring sites during the 2018 Camp Fire in northern California.

Table S1. Median response of each monitor deployed at LBNL relative to co-located TEOM, statistics for 4h averages and event-integrated ratio.

4-h mean data	GRM	AVP	PAI	AQE	ELI	pDR	DT
10th	1.30	1.89	2.79	2.77	1.87	2.21	4.26
25th	1.24	1.78	2.60	2.34	1.77	2.11	4.11
Median	1.17	1.69	2.37	2.18	1.67	1.89	3.99
75th	1.10	1.57	2.15	1.94	1.50	1.69	3.81
90th	1.06	1.32	2.04	1.76	1.37	1.23	3.57
Min	0.99	0.99	1.71	1.51	1.26	1.03	3.29
Max	1.61	2.37	3.50	5.40	2.19	2.33	4.82
RSD (%)	0.11	0.06	0.08	0.06	0.08	0.04	0.13
Event-integrated	1.17	1.68	2.40	2.12	1.58	1.86	3.76

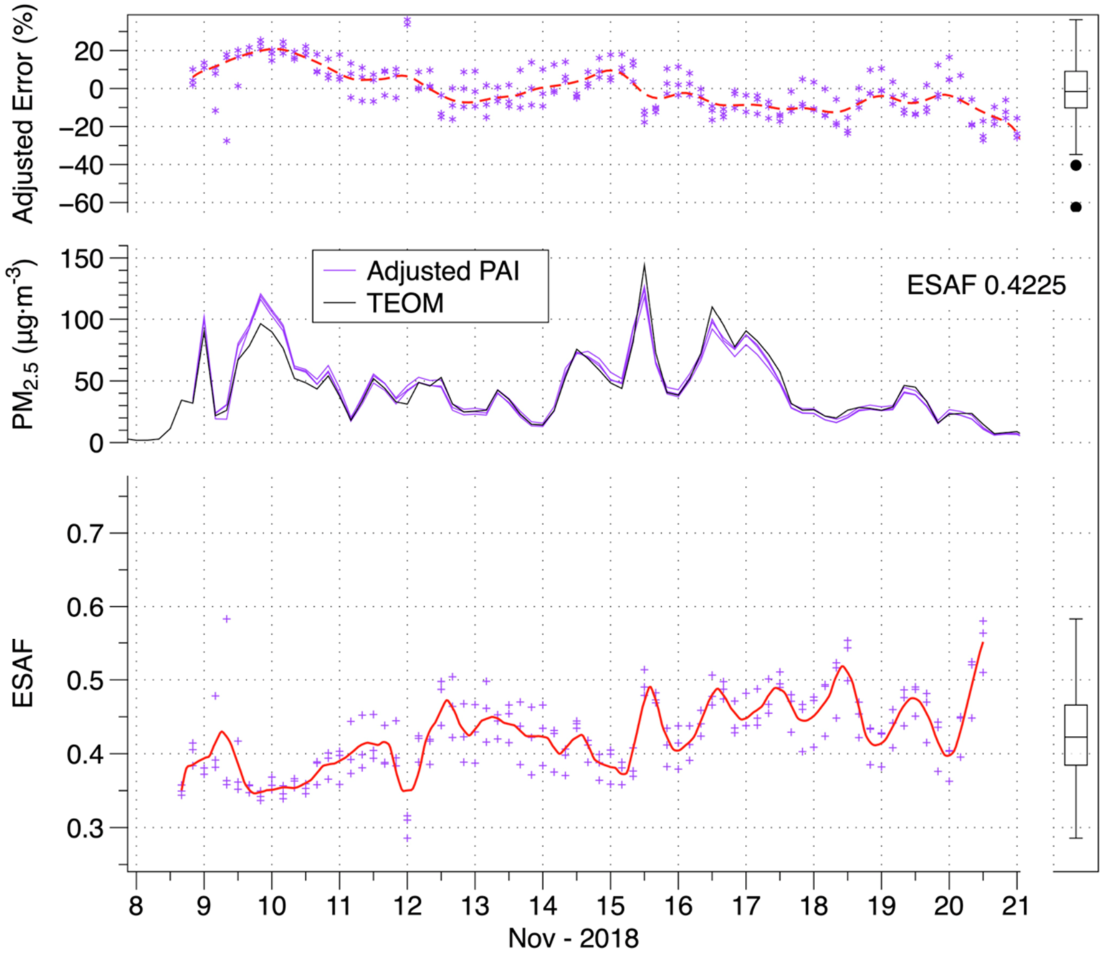

Figure S5. Adjustment factors, adjusted data and error of adjusted data (relative to co-located TEOM $PM_{2.5}$) for 4-h average PAI measurements in in a naturally ventilated lab over 13 days of elevated smoke from the Camp Fire.

Table S2. Sensor data used to calculate adjustment factors for quantifying infiltrating wildfire smoke $PM_{2.5}$ using 4-h means from PurpleAir PA-II monitors nearby to northern California regulatory air quality monitoring stations during the three western US wild fires.

City	AQS Name	Fire	Distance (km)	Mean $PM_{2.5}$ (g m^{-3})	Nearby PA-II units	Valid sensors
Sacramento	1309 T Street	Camp	133	134	11	20
Davis	UCD Campus	Camp	137	82	3	6
Vallejo	Vallejo	Camp	192	92	7	13
Concord	Concord	Camp	206	87	3	4
San Pablo	Rumrill	Camp	210	93	4	8
San Rafael	San Rafael	Camp	213	89	4	8
Berkeley	Aquatic Park	Camp	219	93	3	6
Oakland	Oakland - West	Camp	224	94	3	6
Oakland	Laney College	Camp	226	91	1	2
San Francisco	SF-Arkansas Street	Camp	232	93	4	7
Redwood City	Redwood City	Camp	258	74	5	10
San Jose	San Jose - Knox Ave	Camp	270	80	5	7
Red Bluff	Red Bluff - Walnut St	Carr/ MC	50	52	1	2
Spanish Fork	Spanish Fork (SF)	Pole Creek	23	42	1	2

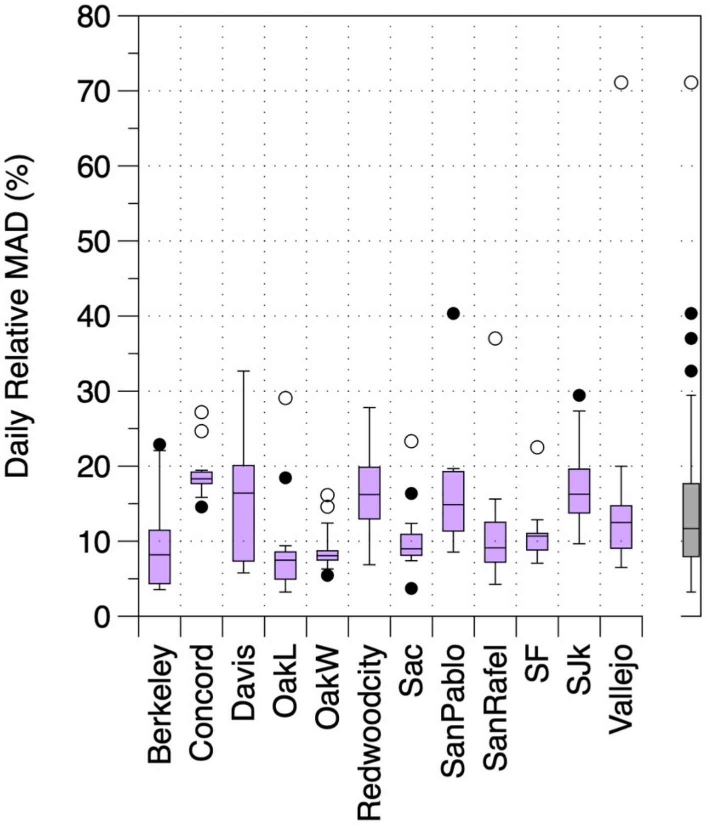

Figure S6. Distributions of daily relative median absolute deviations

The median absolute deviation is a robust measure of the variability in a univariate sample of quantitative data. It is defined as

$$MAD = \text{median}\left(\left|X_i - \tilde{X}\right|\right)$$

where

$$\tilde{X} = \text{median}(X_i)$$

and the relative mean absolute deviation is defined as

$$relative\ MAD = {}^{MAD}/_{\tilde{X}}$$

The relative MAD can be interpreted as the variability in the daily AFs.

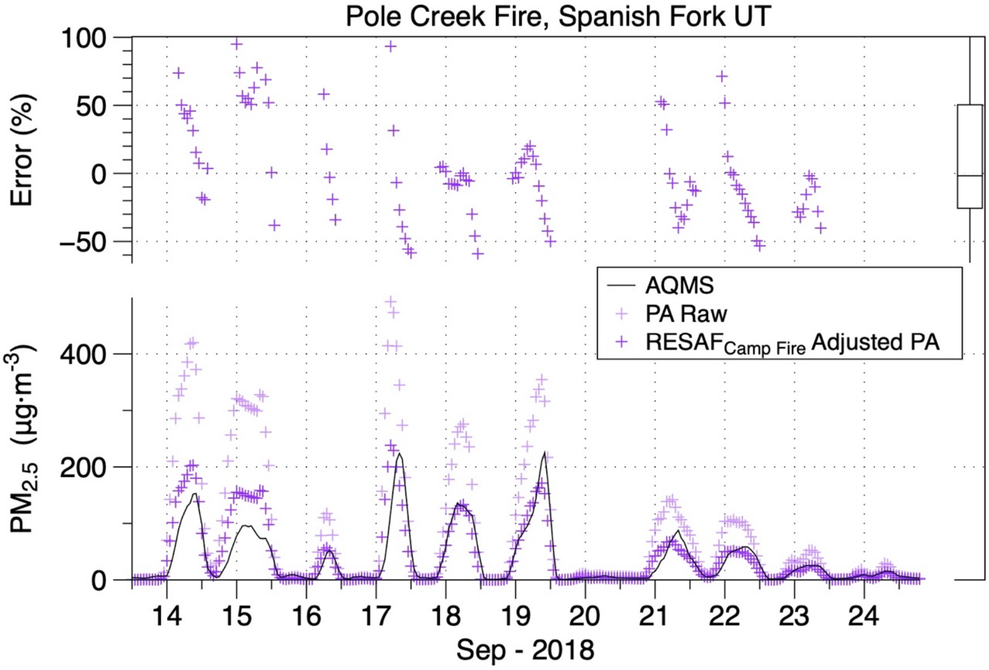

Figure S7. Running 4hr average data for the Pole Creek Fire at Spanish Fork UT. Note the strong diurnal pattern in the PM values. Northern California RESAF applied to PA values when smoke was present (PM>20), this adjustment was appropriate for the Pole Creek fire.

DT and PDR calibration notes

The DT used for this study was factory calibrated approximately 14 months before use. The unit was used in another field study deployed outdoors for approximately 1 week in two different locations in the California Central Valley. Three weeks before the Camp Fire it was deployed in Bakersfield, and one week before the Camp Fire it was in Davis. Over the deployment period the ratio of the average DT reading to the average nearby AQS was 1.87 for Bakersfield and 1.88 for Davis. This is within the range of values in TSI application note EXPMN-007.

The PDR used factory calibrated 33 months before use. Just after this study it was cross-checked with another PDR that was calibrated 21 months before. The cross-calibration involved burning an incense stick and both instruments agreed with differences of less than 1% of the reported values.

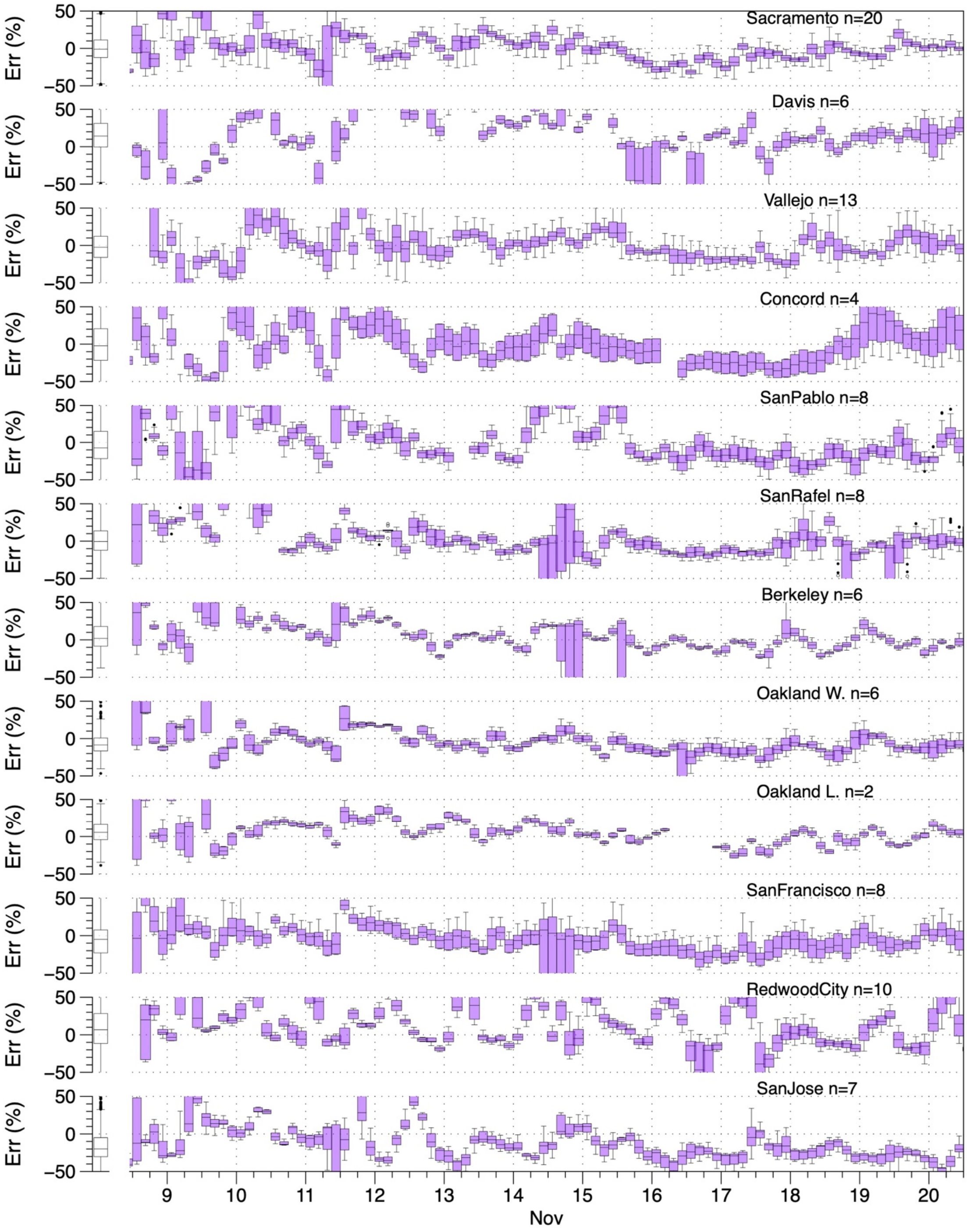

Figure S8. Error distributions for 4 h average data after applying Camp Fire event-specific regional adjustment factor to PA-II monitors near air quality monitoring stations in Northern California.

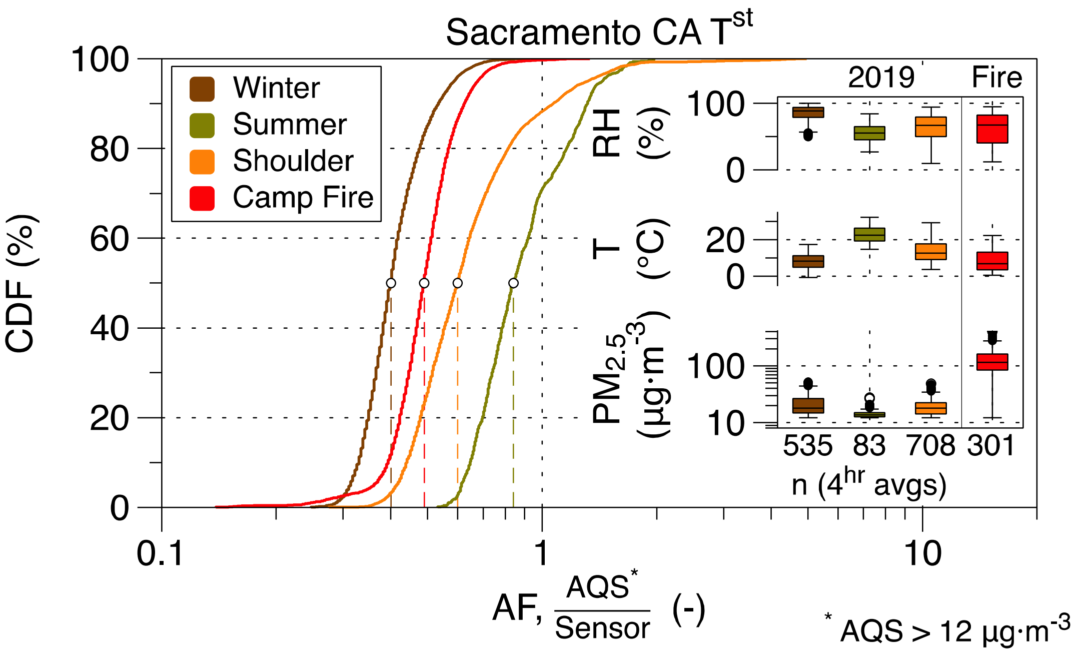

Figure S9. Environmental conditions and PurpleAir II adjustment factors by season over running 4-h intervals with $PM_{2.5}$ above 12 g/m^3 at the T-street air quality monitoring station in Sacramento, CA.

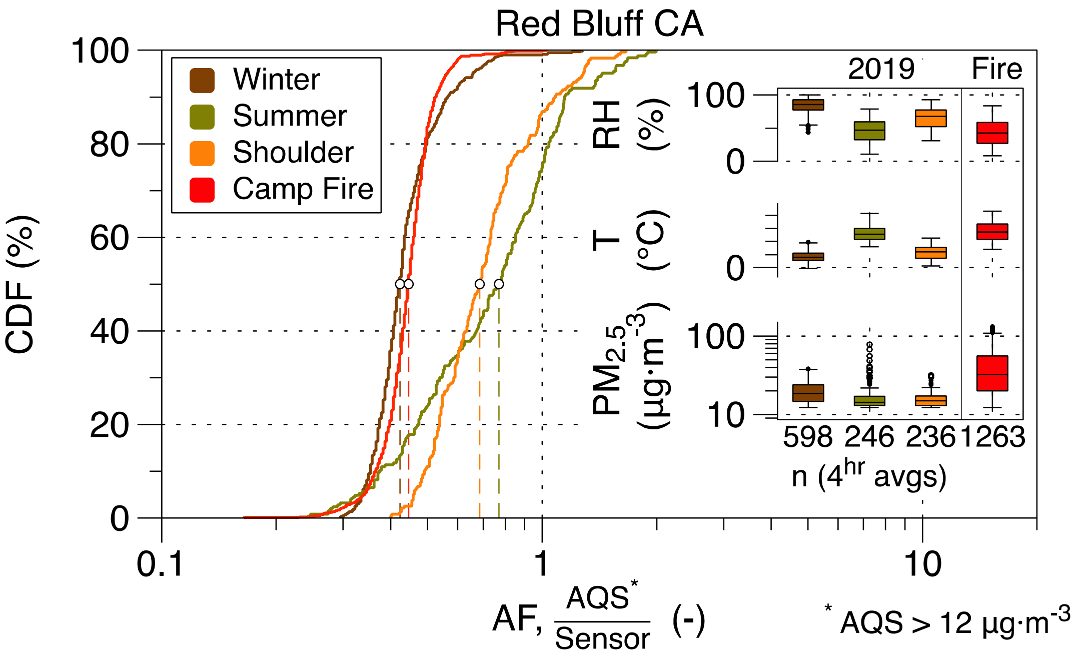

Figure S10. Environmental conditions and PurpleAir II adjustment factors by season over running 4-h intervals with $PM_{2.5}$ above 12 g/m^3 at the air quality monitoring station in Red Bluff, CA.

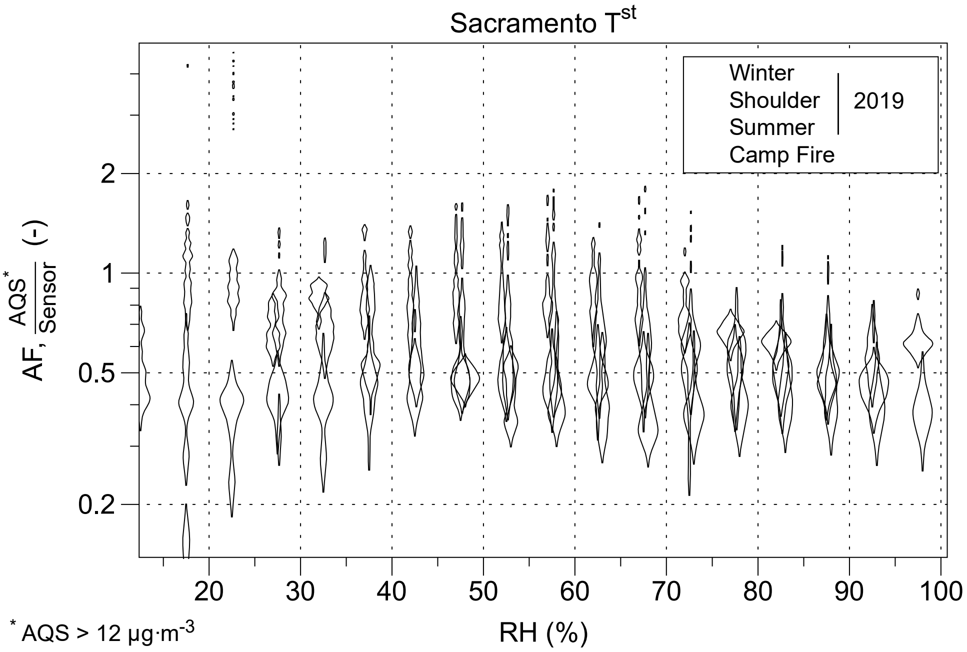

Figure S11. PurpleAir II adjustment factors and relative humidity by season over running 4-h intervals with $PM_{2.5}$ above 12 g/m^3 at the T-street air quality monitoring station in Sacramento, CA.

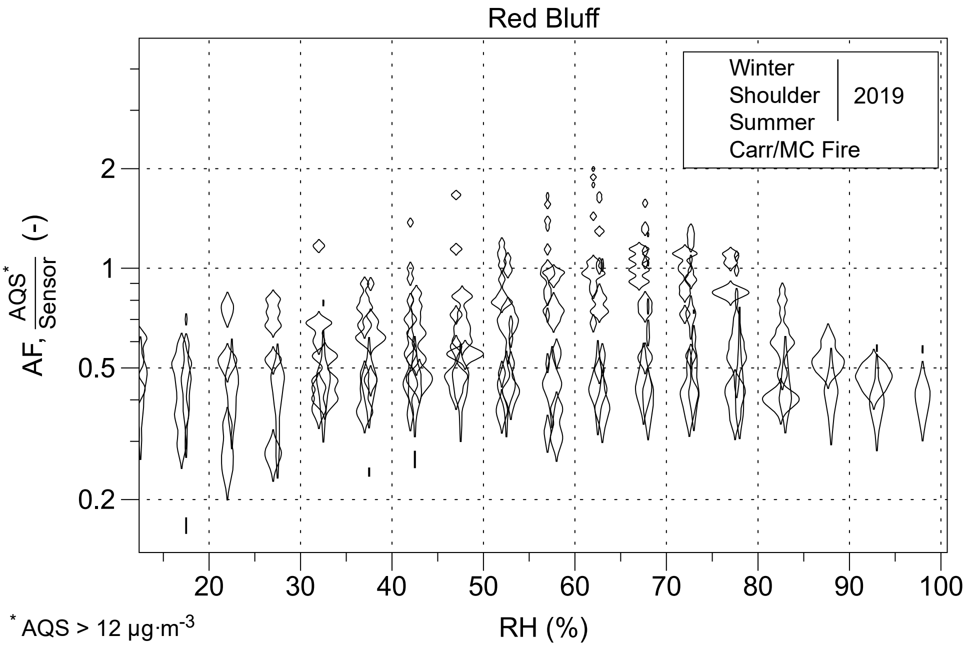

Figure S12. PurpleAir II adjustment factors and relative humidity by season over running 4-h intervals with $PM_{2.5}$ above 12 g/m^3 at the air quality monitoring station in Red Bluff, CA.
